# System-Wide Inpatient Portal  Implementation: Survey of Health Care Team Perceptions

**DOI:** 10.2196/medinform.7707

**Published:** 2017-09-14

**Authors:** Jennifer L Hefner, Cynthia J Sieck, Daniel M Walker, Timothy R Huerta, Ann Scheck McAlearney

**Affiliations:** ^1^ Department of Family Medicine The College of Medicine The Ohio State University Columbus, OH United States; ^2^ Department of Biomedical Informatics The College of Medicine The Ohio State University Columbus, OH United States; ^3^ Division of Health Services Management and Policy The College of Public Health The Ohio State University Columbus, OH United States

**Keywords:** patient portals, hospitalization, medical informatics, patient participation

## Abstract

**Background:**

Inpatient portals, a new type of patient portal tailored specifically to the hospital setting, can allow patients to access up-to-date health information and exchange secure communications with their care team. As such, inpatient portals present an opportunity for patients to increase engagement in their care during a time of acute crisis that emphasizes focus on a patient’s health. While there is a large body of research on patient portals in the outpatient setting, questions are being raised specifically about inpatient portals, such as how they will be incorporated into the flow of patient care in hectic, stressed, team-based hospital settings.

**Objective:**

Our aim is to improve understanding about hospital care team members’ perceptions of the value of an interactive patient portal for admitted patients, as well as to ascertain staff orientation toward this new technology.

**Methods:**

Throughout the course of 2016, an inpatient portal, MyChart Bedside (MCB) was implemented across a five-hospital health system. The portal is a tablet-based app that includes a daily schedule, lab/test results, secure messaging with the care team, a place to take notes, and access to educational materials. Within a month of initial rollout, hospital care team members completed a 5-minute, anonymous online survey to assess attitudes and perceptions about MCB use and staff training for the new technology.

**Results:**

Throughout the health system, 686 staff members completed the survey: 193 physicians (23.6%), 439 nurses (53.7%), and 186 support staff (22.7%). Questions about the importance of MCB, self-efficacy in using MCB with patients, and feelings about sufficient training and resources showed that an average of 40-60% of respondents in each group reported a positive orientation toward the MCB technology and training received. This positive orientation was highest among support staff, lower among nurses, and lowest for physicians (all differences by staff role were statistically significant at *P*<.001). Additionally, 62.0% of respondents reported “not enough” training.

**Conclusions:**

Despite the robust training effort, similar to that used in previous health information technology implementations at this health system, hospital care team members reported only a moderately positive orientation toward MCB and its potential, and the majority wanted more training. We propose that due to the unique elements of the inpatient portal—interactive features used by patients and providers requiring explanation and collaboration—traditional training approaches may be insufficient. Introduction of the inpatient portal as a new collaborative tool may thus require new methods of training to support enhanced engagement between patients and their care team.

## Introduction

Patient portals—a class of electronic personal health records (PHRs) tethered to an electronic health record (EHR)—allow patients to view lab and medication information, schedule appointments, and exchange secure messages with providers [1]. Growth in the availability of patient portals has been almost exclusively limited to the ambulatory environment, with studies linking portal use to improved self-management of chronic conditions [2-6] as well as providing evidence of their potential to improve health and lower costs [7-9]. Inpatient portals are emerging as a new type of patient portal tailored specifically to the hospital setting and offer the opportunity for patients to increase engagement with the portal during a time of acute crisis that emphasizes focus on a patient’s health.

As portals are interactive, provide up-to-date health information for patients, and enable secure communications with their care team, questions are being raised specifically about inpatient portals such as how they will be incorporated into the flow of patient care in hectic, stressed, team-based hospital settings [9]. Research on inpatient portals, however, is scant, with fewer than 10 studies published that have examined patient use and acceptance in small-scale implementations of inpatient portal technologies [10-13]. Further, while initial studies have reported generally positive findings related to inpatient portal use, the technologies studied have not included interactive elements such as secure messaging with the care team. A recent case study of inpatient portal use at five different academic medical centers, for instance, found variation in the availability of portal features, emphasizing the need to study these novel, interactive elements of inpatient portals [9].

MyChart Bedside (MCB), an inpatient portal, is a tablet-based app patients can use to access their data while admitted at an Epic-equipped hospital and includes interactive functionalities. MCB was developed by Epic—a proprietary software company whose EHR has been adopted by hospitals serving more than 50% of US patients—to provide patients and their families and caregivers access to information customized to the inpatient setting. It includes a daily schedule, lab/test results, secure messaging with the care team, a place to take notes, and access to educational materials. Recent implementation of MCB across a large Midwestern multihospital health system provided the opportunity to survey staff during the initial implementation phase to explore the perceived value of a patient portal for admitted patients from the clinician perspective, as well as to ascertain staff attitudes to deployment of this new technology. This study adds to what we expect will be a growing literature that identifies the unique dynamics associated with inpatient use of patient portals.

## Methods

### Study Setting

Throughout 2016, MCB was implemented across all units of a five-hospital tertiary care academic medical center in a large metropolitan city, with nearly 1400 inpatient beds and over 5000 providers. The MCB implementation was accompanied by a training and engagement plan that included identifying and training unit “champions” who received dedicated time to devote to this role, delivering information sessions on each unit to orient staff to the technology and tablet provisioning plan, having information technology staff available on the units during the initial “go-live”, and providing access to online documentation detailing tablet provisioning procedures and e-learning modules focused on MCB.

### Survey Process

We surveyed hospital staff across the health system to assess attitudes and perceptions about MCB use and their training to use the new technology. Specifically, within a month of initial MCB implementation, hospital care team members received a recruitment email with a link to the survey. Over the implementation timeframe, this email was sent to all 5000 providers through unit-specific listservs. This protocol was approved by the study site’s institutional review board.

### Survey Instrument

The anonymous, online survey instrument took about 5 minutes to complete. Questions included the respondent’s role within the academic medical center (physician, nurse, unit clerical associate [UCA], patient care assistant [PCA]), and a series of questions about the respondent’s orientation toward and training with the technology, such as the importance of MCB, self-efficacy for using MCB, and feelings about sufficient training and resources (5-point response categories from “Strongly Disagree” to “Strongly Agree”). In addition, the seven features of MCB were listed—Dining on Demand, Education, Secure Messaging, Medication List, Problem List, Schedule, and Description of Care Team—with respondents asked to rate “the features of MCB according to how much you expect that patients will use them” (5-point response categories: “Not at all” to “A lot”) and “the features of MCB according to how much you expect that patients will benefit from them” (5-point response categories: “Not at all” to “Extremely”).

### Survey Analysis

Descriptive statistics were calculated for each survey question and when appropriate, cross tabulations and chi square tests of a statistically significant difference between categories were conducted to compare response choices between survey groups. For these analyses, 5-point response categories listed in the above section were also collapsed into a binary variable equal to 1 for the top two response categories (eg, “Agree/Strongly agree”). Additionally, the UCA and PCA respondent groups were collapsed into one job category due to small numbers and the fact that they played similar roles in the inpatient medical care hierarchy. This job category is referred to as “clinical support staff” below.

## Results

Across the health system, 686 staff members completed the survey: 193 physicians (23.6%), 439 nurses (53.7%), and 186 clinical support staff (22.7%). Table 1 presents responses to questions about respondents’ orientation toward MCB. We found that the questions about the importance of MCB, self-efficacy in using MCB, and feelings about sufficient training and resources showed an average of 40-60% of respondents in each group reporting a positive orientation toward the MCB technology and training received. This positive orientation was highest among support staff, lower among nurses, and lowest for physicians (all differences by staff role were statistically significant at *P*<.001). On average, 62.0% (425/686) of respondents reported “not enough” training. Among physicians, 79.9% (154/193) responded they had lacked sufficient training compared with 61.7% (271/439) of nurses and 46.2% (86/186) of support staff.

When asked about the MCB features patients would be likely to use most often and how much patients would benefit, respondents reportedly valued the features differently (Table 2). Dining on Demand was the feature respondents reported patients would most likely use and benefit from, with more than two thirds of respondents reporting patients would use electronic meal ordering “A lot/Often” (473/686, 68.9%) and would benefit “Very much/Extremely” (449/686, 65.5%). Next, almost half of respondents reported the Medication List and the Schedule as features patients were likely to both use and benefit from. Secure Messaging was less frequently endorsed, with low rates of likely use and benefit: 16.5% (113/686) and 24.9% (171/), respectively. Notably, there was a large discrepancy between perceptions of use and benefit in the Education feature, with 22.4% (154/686) reporting likely patient use and 50.9% (349/686) reporting potential patient benefit.

**Table 1:** Hospital staff perspectives on MCB technology and training^a^.

	Strongly agree/Agree, %			
	All (n=686)	Physicians (n=193)	Nurses (n=439)	Clinical support staff (n=186)
I am aware of the reasons this health system is implementing MCB.	75.2	57.8	78.5	85.2
I feel the health system is promoting use of MCB.	70.6	56.9	73.2	76.5
It is important to provide access to MCB to patients in this hospital.	63.2	48.3	63.4	76.4
I believe that patients will benefit from MCB.	57.0	41.5	55.9	73.9
I understand responsibilities within the care team on my unit for responding to MCB questions.	56.6	33.3	57.6	75.0
I can play an important role in helping patients manage their health through MCB.	47.3	36.1	46.8	58.6
I am interested in helping patients manage their health through MCB.	48.6	38.4	45.4	65.6
There are sufficient resources on my unit to effectively incorporate MCB.	42.4	19.7	43.7	59.5
I have the tools I need to help my patients use MCB.	40.2	22.5	39.3	58.6

^a^For all statements, differences between groups were statistically significant at *P*<.001.

**Table 2:** Hospital staff ratings of MCB features patients are likely to use most often and how much patients will benefit from them.

	Patients will use “A lot/Often”, %	Patient will benefit “Very/Extremely”, %
Dining on Demand	68.9	65.5
Education	22.5	50.9
Secure Messaging	16.6	25.0
Medication List	41.4	43.9
Problem List	21.9	29.7
Schedule	37.7	43.0
Description of Care Team	27.3	37.7

## Discussion

### Principal Considerations

Results from this early implementation survey revealed staff had a moderately positive orientation toward the MCB tool and its potential, and this varied by job role. Clinical support staff (PCAs and UCAs) was most positively oriented toward the technology, while nurses and physicians were less convinced that MCB was an important tool. Further, physicians were less confident than the other groups about both their role with the technology and whether they had sufficient training to feel comfortable incorporating MCB in their workflow. As physicians at this institution were less involved in the training and implementation than other groups, this may account for their less positive attitude toward the technology. The primary physician use of MCB is secure message communication, a new feature in the hospital setting, thus their lack of comfort with MCB suggests the need for physician training focused on this feature.

Our findings about less positive nurse feelings are less clear. Although MCB provisioning procedures differ by unit across the health system, the nurses and support staff are all involved in the distribution, use, and collection of MCB. Increasing engagement of nurses involved in direct patient care has been highlighted as an important element of portal use in the inpatient setting [9]. Given that nurses can be expected to interact with patients frequently using the portal, whether by responding to questions when they are in the patient’s room or via secure messages, our findings suggest that additional focus on improving nurse perceptions of this tool may be important.

### Limitations

This study has several notable limitations. First, given the survey was anonymous, we do not have any information about nonrespondents. There could have been nonresponse bias related to satisfaction with this new technology. For example, those with more negative attitudes may have been more likely to not respond. If this is the case, then the true level of negative feelings is even lower. It is also possible that demographic factors such as age and gender, tenure at the organization, or experience in the field may play roles influencing attitudes toward inpatient portals. This short paper is the first reporting results from a program of research for this study team on the implementation and use of an inpatient portal across a large medical center. Interviews with staff and providers are ongoing and will provide crucial information about the facilitators and barriers to improving providers’ attitudes toward, and increasing their confidence using, this new technology.

### Conclusions

For this implementation of MCB, the medical center engaged in a robust staff education effort similar to that used in previous health information technology (HIT) implementations. This general approach, however, may not account for unique features of an inpatient portal compared to other hospital-focused HIT tools. First, the inpatient portal includes features utilized by patients, not just the care team, and these features may require additional explanation to support their appropriate use. Second, the inpatient portal introduces the ability to communicate via secure messaging and represents a new avenue for collaboration between the patient and the care team not previously available in the inpatient setting. Research in the outpatient context suggests that this type of collaboration is particularly challenging for both patients and providers because it requires new rules by which each party engages [14]. Introduction of the inpatient portal as a new collaborative tool may thus require new methods of training to support enhanced engagement between patients and their care team.

## Abbreviations

EHR: electronic health record
HIT: health information technology
MCB: MyChart Bedside
PCA: patient care associate
PHR: personal health record
UCA: unit clerical associate
